# Protective effect of pinocembrin on zearalenone-induced hepatotoxicity via PI3K/AKT and NRF2 signaling in albino mice

**DOI:** 10.55730/1300-0152.2795

**Published:** 2026-02-05

**Authors:** Sujatha TEJAVAT

**Affiliations:** Division of Pathology, Department of Biomedical Sciences, College of Medicine, King Faisal University, Al-Ahsa, Saudi Arabia

**Keywords:** Zearalenone, pinocembrin, liver, NRF2, apoptosis

## Abstract

**Background/aim:**

Zearalenone (ZEA) is a Fusarium-derived mycotoxin that frequently contaminates food and feed and induces hepatotoxicity in humans and animals through oxidative stress and inflammation. Pinocembrin (PCM), a naturally occurring flavonoid, has potent antioxidant and antiinflammatory properties. This study investigated the hepatoprotective effects of PCM against ZEA-induced liver injury in albino mice, with a focus on the nuclear factor erythroid 2–related factor 2 (NRF2) and phosphoinositide 3-kinase/protein kinase B (PI3K/Akt) signaling pathways.

**Materials and methods:**

Twenty-four albino male mice were allocated into four groups: (1) control (vehicle 0.05% dimethyl sulfoxide), (2) ZEA-treated (40 mg/kg body weight), (3) PCM-treated (50 mg/kg body weight), and (4) ZEA + PCM. After 42 days of oral treatment, serum alanine aminotransferase, aspartate aminotransferase, and alkaline phosphatase levels were determined. Hepatic oxidative stress markers were assessed. Liver histopathology was evaluated using standard staining techniques. Western blot analysis was performed to determine the expression of several proinflammatory cytokines signaling proteins: interleukin-6, interleukin-1β, tumor necrosis factor-α, NRF2 pathway components, apoptosis-related proteins (B-cell lymphoma 2 [Bcl-2], cleaved caspase-3), and PI3K/Akt.

**Results:**

ZEA administration caused significantly elevated serum liver enzyme and hepatic malondialdehyde levels while causing a reduction in other oxidative stress markers. Histopathological examination revealed marked hepatic architectural disruption and cellular degeneration in the ZEA-treated group. Additionally, ZEA caused downregulation of antiapoptotic signaling proteins (such as Bcl-2) and upregulation of proinflammatory cytokines and cleaved caspase-3 expression. The coadministration of PCM facilitated markedly attenuated ZEA-induced biochemical, oxidative, inflammatory, apoptotic, and histopathological alterations. PCM treatment restored antioxidant enzyme activities and produced significant enhancement of NRF2/HO-1/NQO1 and PI3K/Akt signaling.

**Conclusion:**

PCM effectively protects against ZEA-induced hepatotoxicity in albino mice by mitigating oxidative stress, inflammation, and apoptosis. These protective effects are partially mediated through activation of the NRF2 signaling pathway and modulation of the PI3K/Akt pathway.

## 1. Introduction

Many *Fusarium* species produce zearalenone (ZEA), a nonsteroidal estrogenic mycotoxin that commonly contaminates maize, wheat, and barley worldwide. The widespread presence of ZEA in the food and feed chain poses a serious threat to both human and animal health (Rajendran et al., 2020; Majer-Baranyi et al., 2021). ZEA exposure has been associated with reproductive toxicity, immunotoxicity, and hepatotoxicity (AbuZahra et al., 2021). Due to the liver’s essential function in metabolism and detoxification, hepatic injury is a major concern. The mechanisms underlying ZEA-induced hepatotoxicity involve oxidative stress, inflammation, and apoptosis. During hepatic metabolism of ZEA, excessive production of reactive oxygen species (ROS) disrupts the balance between prooxidant and antioxidant defense systems (Li et al., 2021; Liu et al., 2023). Oxidative stress can lead to lipid peroxidation, DNA damage, and protein dysfunction, ultimately resulting in hepatocellular injury. Moreover, ZEA exposure stimulates the release of proinflammatory cytokines, such as interleukin-6 and interleukin-1β (IL-6 and IL-1β, respectively) and tumor necrosis factor-α (TNF-α), thereby exacerbating liver inflammation and tissue damage. Apoptotic signaling further accelerates hepatic injury through upregulation of proapoptotic proteins, including cleaved caspase-3, and downregulation of antiapoptotic proteins such as B-cell lymphoma-2 (Bcl-2).

In recent years, antioxidant and antiinflammatory natural substances have been investigated as protective agents against mycotoxin-induced cell and tissue damage (Qi et al., 2022; Chellappan and Peramaiyan, 2024). Honey, propolis, and various plants contain a considerable amount of the flavonoid pinocembrin (PCM). PCM possesses antioxidant, antiinflammatory, neuroprotective, and anticancer properties (Alzahrani and Rajendran, 2021). The antioxidant effects of PCM are largely attributed to its ability to scavenge free radicals and modulate endogenous antioxidant enzymes. In addition, PCM suppresses the production of proinflammatory mediators, suggesting a potential role in mitigating inflammation-associated tissue injury (Gu et al., 2017; Zhang et al., 2022; Ali et al., 2024). The transcription factor called nuclear factor erythroid 2-related factor 2 (NRF2) protects cells from oxidative damage and inflammation (Sun et al., 2018; Wu et al., 2022). After activation, NRF2 translocates to the nucleus and binds to antioxidant response elements (ARE), leading to the upregulation of cytoprotective genes such as heme oxygenase-1 (HO-1) and nicotinamide adenine dinucleotide (NAD(P)H) quinone dehydrogenase 1 (NQO1). These enzymes play a key role in cellular detoxification and protection against oxidative damage protection. Activation of the NRF2 signaling pathway may protect against toxicological insults, including mycotoxin-induced organ damage (Bosaad et al., 2024). The phosphoinositide 3-kinase/protein kinase B (PI3K/Akt) signaling pathway is another important intracellular signaling cascade involved in cell survival, proliferation, and inflammation (Ammar et al., 2025). Emerging evidence suggests a functional interaction between the PI3K/Akt and NRF2 pathways in enhancing cellular antioxidant defense mechanisms.

Therefore, the present study aimed to investigate the protective effects of PCM against ZEA-induced hepatotoxicity in albino mice. Specifically, the study evaluated the effects of PCM on serum liver enzyme activities, hepatic oxidative stress markers, and liver histopathology. Furthermore, the underlying molecular mechanisms were explored by assessing the expression of inflammatory cytokines (IL-6, IL-1β, and TNF-α) and apoptosis-related proteins (Bcl-2 and cleaved caspase-3), as well as the involvement of the NRF2 signaling pathway (NRF2, HO-1, and NQO1) and its potential interaction with the PI3K/Akt pathway.

## 2. Material and methods

ZEA (CAS No.: 17924-92-4) and PCM (CAS No.: 480-39-7) were purchased from Sigma (St. Louis, USA). All other chemicals were of analytical grade. Primary and secondary antibodies were purchased from Thermo Fisher Scientific, Inc. (USA). The antibody against cleaved caspase-3 was obtained from Cell Signaling Technology (USA). The β-actin antibody was used at a dilution of 1:5000. Detailed information for all antibodies, including catalog numbers and dilutions, is provided in the Supplementary File (Table S).

### 2.1. Animal experiments

#### 2.1.1. Animals

Six-week-old male Swiss albino mice (20 ± 5 g) were obtained from the King Saud University Experimental Animal Care Center (KSUEACC), Riyadh, Saudi Arabia. The mice (n = 24) were randomly allocated into four groups of six individuals per group. The animals were maintained in standard cages under controlled conditions: temperature of 25 ± 2 °C, humidity of 50%–60%, and a 12-h light/dark cycle (Percie du Sert et al., 2020). Standard rodent food and water were available ad libitum. Prior to the experiment, the mice underwent a one-week acclimatization period within the laboratory. All animal welfare and experimental protocols were approved by King Faisal University’s Animal Research Ethics Committee (reference number EA0013245).

### 2.2. Experimental design and treatments

The mice were randomly allocated into four experimental groups (n = 6 per group). All treatments were conducted over a six-week (42-day) experimental period. In group I (control), the mice received the vehicle only (0.05% dimethyl sulfoxide [DMSO]) orally once daily for 42 days. In group II (ZEA), the mice were administered ZEA (40 mg/kg body weight) three times per week via subcutaneous injection as previously described (Rajendran et al., 2020; AbuZahra et al., 2021). In group III (ZEA + PCM), the mice received the same ZEA dosage as in group II but were also treated with PCM (50 mg/kg body weight) via oral gavage once daily. Mice in Group III received both treatments on the same day; PCM was administered 3 h after each ZEA injection. In group IV (PCM), the mice were given oral PCM at a dose of 50 mg/kg body weight once daily (Gong et al., 2020; Alzahrani and Rajendran, 2021).

#### 2.2.1. Sample collection

At the end of the six-week experimental period, all mice were euthanized via inhalation of 4% isoflurane in oxygen, in accordance with the *Guide for the Care and Use of Laboratory Animals* (National Research Council Committee, 2011; van der Wijngaart et al., 2023). Blood and liver tissues were taken, snap frozen to halt metabolic activities, and preserved at −80 °C for later examination (van der Wijngaart et al., 2023). Relative liver weight was measured to evaluate the effect of PCM on ZEA-induced alterations in liver tissue mass.

#### 2.2.2. Biochemical assays

Biochemical analyses were performed using serum obtained from the blood samples. The activities of aspartate aminotransferase (AST), alanine aminotransferase (ALT), alkaline phosphatase (ALP), lactate dehydrogenase (LDH), IL-6, and TNF-α were measured using commercially available kits according to the manufacturer’s instructions (Spectrum Diagnostics, Obour, Egypt). The liver tissue antioxidant enzyme activities of glutathione peroxidase (GPx), total superoxide dismutase (SOD,) and catalase (CAT) were quantified using assay kits obtained from Cayman Chemical (USA).

#### 2.2.3. Measurement of MDA

Malondialdehyde (MDA) levels in the liver tissues of ZEA-exposed mice with or without PCM treatment were assessed based on a colorimetric reaction. The intensity of the resulting color is proportional to the MDA concentration and was quantified spectrophotometrically at 532 nm. Results were expressed as nmol MDA/mg protein.

#### 2.2.4. Western blot

Following the experimental treatments, liver tissues were harvested and washed once using ice-cold phosphate-buffered saline. Total tissue protein extracts were prepared according to a previously established protocol. Protein concentrations were determined using a Bio-Rad protein assay kit using bovine serum albumin as the standard. Subsequently, 50 μg of protein from each sample were subject to electrophoresis on sodium dodecyl sulfate-polyacrylamide gradient gels (12%). The separated proteins were then transferred onto nitrocellulose membranes overnight. To minimize nonspecific antibody binding, the membranes were blocked by incubating them in 5% skimmed milk at 37 °C for 30 min, followed by incubation with the appropriate primary antibodies for 2 h. After washing, the membranes were incubated with horseradish peroxidase-conjugated goat antimouse or antirabbit secondary antibodies for 1 h. Protein bands were visualized using an enhanced chemiluminescence substrate (Pierce Biotechnology, Rockford, IL, USA) and imaged with a LI-COR C-Digit Blot Scanner. Densitometric quantification of the protein bands was performed using Image Studio Lite (LI-COR Biosciences, Lincoln, NE, USA) as described by Mahmood and Yang (2012).

#### 2.2.5. Histopathology analysis

After harvest, the liver tissues were preserved in 4% formalin, dehydrated through graded ethanol solutions (70%, 90%, and 100%), and embedded in paraffin wax. Sections (5 μm thick) were cut, deparaffinized with xylene, and stained with hematoxylin and eosin (H&E) for a histopathological evaluation.

### 2.3. Statistics

Quantitative data are presented as mean ± standard deviation. Statistical differences among groups were analyzed using one-way analysis of variance (ANOVA). Significant ANOVA results were further analyzed using Tukey’s post hoc test to determine specific intergroup differences. GraphPad Prism (version 8.2) was used for all statistical analyses. A p value < 0.05 was considered statistically significant.

## 3. Results

### 3.1. The effect of PCM on liver marker enzymes

**Figure 1:**
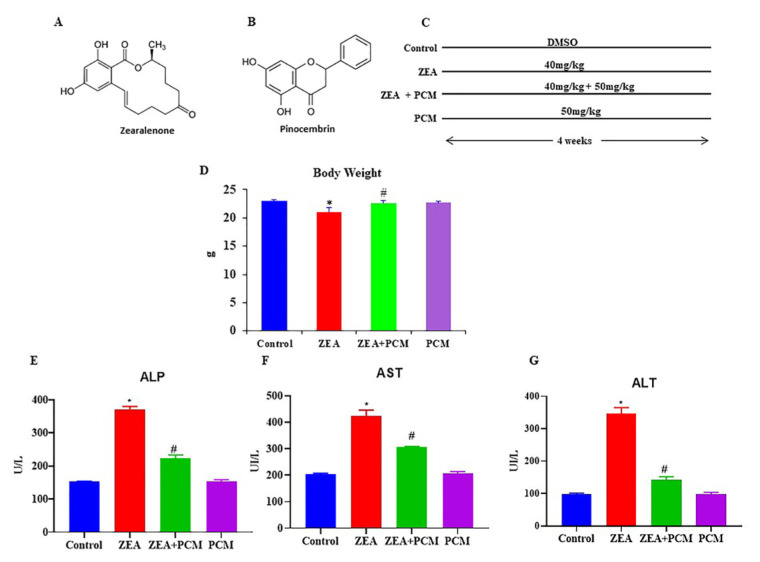
The effect of PCM on body index and hepatic markers. (A) chemical structure of zearalenone (ZEA), (B) chemical structure of PCM, (C) animal experimental design, (D) body weight (g), and (E–G) liver markers alkaline phosphatase (ALP), aspartame aminotransferase (AST), and alanine aminotransferase (ALT) expressed as U/L. Significant differences compared to the control group are indicated by * (p < 0.05). Significant differences comparing the ZEA and PCM + ZEA treatment groups are indicated by # (p < 0.05).

Figure 1 shows the chemical structures of ZEA and PCM, the impact of ZEA and PCM on body weight, and the overall experimental design. Exposure to ZEA did not significantly affect body weight in comparison to the control group. The combination of ZEA and PCM led to a modest, yet statistically significant, increase in body weight (p < 0.05). The PCM therapy alone had no impact on body weight. The influence of ZEA and PCM on hepatic function was evaluated by quantifying the levels of the liver enzymes ALP, AST, and ALT (Figure 1). Exposure to ZEA caused a marked elevation in the levels of all three enzymes (p < 0.05), which indicates hepatic injury. Coadministration of PCM with ZEA caused a significant reduction in the ZEA-induced increase in ALP, AST, and ALT levels (p < 0.05). PCM alone did not markedly influence the levels of these enzymes.

### 3.2. The effect of PCM on inflammatory cytokines in serum

**Figure 2:**
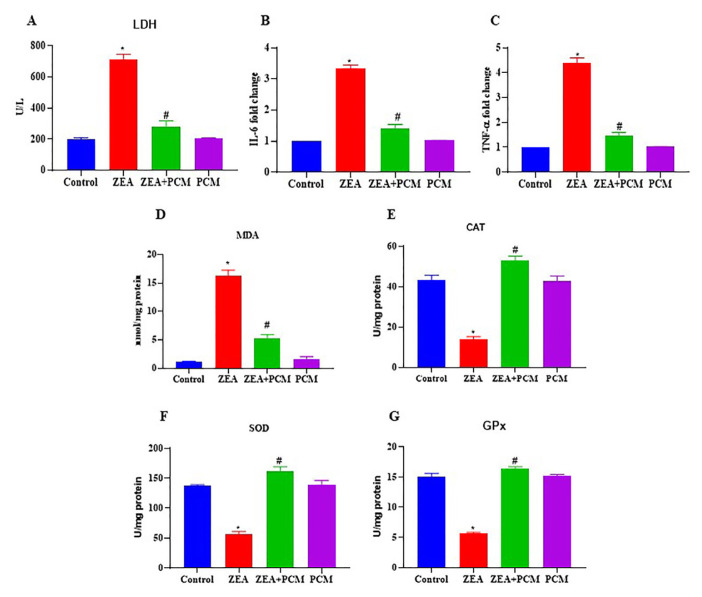
The effect of PCM on oxidative stress markers. (A) serum lactate dehydrogenase (LDH, expressed as U/L), (B) serum tumor necrosis alpha (TNF-α), (C) serum interleukin 6 (IL-6), (D) liver malondialdehyde (MDA) levels expressed as nmol/mg protein, (E) liver catalase (CAT) activity expressed as U/mg protein, (F) liver superoxide dismutase (SOD) activity expressed as U/mg protein, and (G) liver glutathione peroxidase (GPx) activity expressed as U/mg protein. Significant differences compared to the control group are indicated by * (p < 0.05). Significant differences comparing the ZEA and PCM + ZEA treatment groups are indicated by # (p < 0.05).

Levels of the liver enzyme LDH significantly increased in ZEA-treated animals, whereas ZEA with PCM decreased it (Figure 2). TNF-α and IL-6 were evaluated to investigate the protective effect of PCM against ZEA-induced inflammation in mice. The amounts of TNF-α and IL-6 were similar in the control and PCM-only groups (p < 0.05, Figure 2). In comparison to the control groups, the mice with ZEA-induced liver damage exhibited significantly elevated levels of these inflammatory markers. Furthermore, PCM therapy caused a decrease in the levels of TNF-α and IL-6 (Figure 2). The data demonstrate that PCM treatment mitigated inflammation by causing a decrease in the activities of these cytokines.

### 3.3. The effect of PCM on oxidative stress

The antioxidant effects of PCM on ZEA-induced hepatic damage were assessed in liver tissues by quantifying oxidative stress and antioxidant markers, including malondialdehyde (MDA; a lipid peroxidation marker), superoxide dismutase (SOD), catalase (CAT), and glutathione peroxidase (GPx). ZEA treatment caused a significant increase in MDA levels (Figure 2D) while causing a reduction in the activities of CAT, SOD, and GPx (Figure 2) compared to the control group (p < 0.05). The PCM therapy markedly rectified the changes induced by ZEA, restored antioxidant enzyme activity, and caused a reduction in MDA levels (Figure 2). PCM alone did not significantly affect SOD, CAT, or GPx levels. These data demonstrate that PCM protects against ZEA-induced liver damage by mitigating oxidative stress.

### 3.4. The effect of PCM on liver morphology

**Figure 3:**
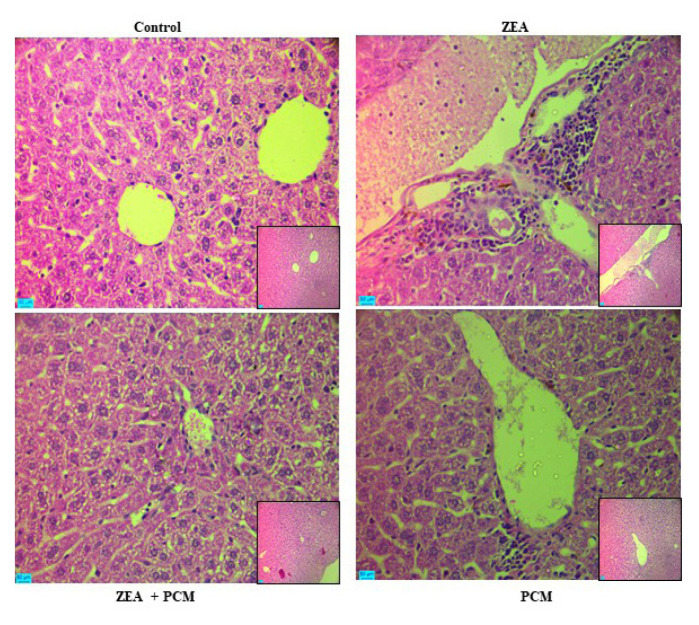
Histopathological analysis of liver tissue. (A) hematoxylin and eosin (H&E) staining and (B) the histopathological grade values of the hepatocyte necrosis and inflammatory cell infiltration. An optical microscope (Leica D6000, Leica, Wetzlar, Germany) was used for the observation and measurements of the prepared sections, and images were acquired at 200× magnification (scale bar = 50 μm).

Histological evaluation of the liver tissues was performed to assess ZEA-induced hepatic damage (Figure 3). H&E-stained sections from the control group revealed preserved hepatic architecture with hepatocytes arranged in single-cell-thick plates radiating from the central vein. In contrast, liver sections from the ZEA-treated group exhibited marked pathological alterations, including hepatocellular nuclear enlargement, increased sinusoidal edema, inflammatory cell infiltration, and intense cytoplasmic staining in enlarged hepatocytes. These histopathological changes were markedly noted in mice that had been cotreated with PCM. Conversely, liver sections from the PCM-only group showed no observable histological abnormalities and were comparable to those of the control group.

### 3.5. The effect of PCM on proinflammatory cytokines in liver tissue

**Figure 4:**
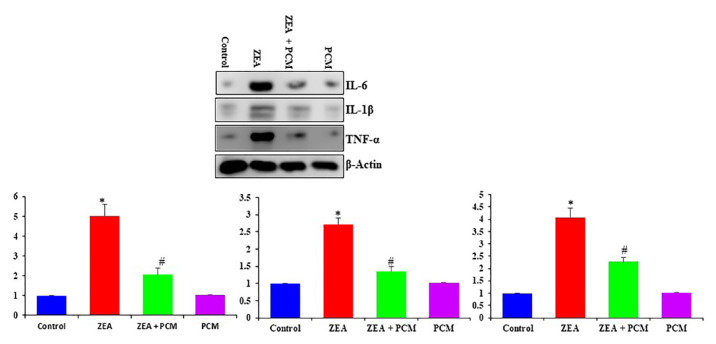
The effect of PCM on liver tissue inflammatory cytokines. Significant differences compared to the control group are indicated by * (p < 0.05). Significant differences comparing the ZEA and PCM + ZEA treatment groups are indicated by # (p < 0.05).

We subsequently examined the effect of PCM on the ZEA-induced expression of the inflammatory cytokines by Western blot analysis. The increased levels of IL-6, IL-1β, and TNF-α caused by ZEA were significantly reduced after PCM treatment, as demonstrated in Figure 4. These tests collectively indicate that PCM inhibits the synthesis of inflammatory mediators and cytokines.

### 3.6. The effect of PCM on PI3K/AKT signaling pathway

**Figure 5:**
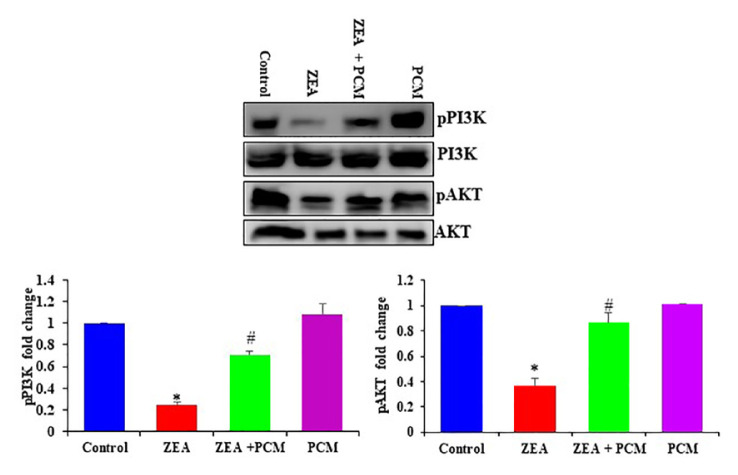
The effect of PCM on liver tissue pPI3K/pAKT signaling, and the total PI3K and AKT without altering total protein expression. Significant differences compared to the control group are indicated by * (p < 0.05). Significant differences comparing the ZEA and PCM + ZEA treatment groups are indicated by # (p < 0.05).

The activation of phosphatidylinositol 3-kinase/protein kinase B (pPI3K/pAKT) in the liver was examined to clarify whether PCM alleviates ZEA-induced hepatic injury via this pathway (Figure 5). ZEA treatment caused a reduction in PI3K/AKT phosphorylation in the liver relative to the control mice. However, the animals treated with PCM showed an increase in pPI3K/pAKT phosphorylation (Figure 5).

### 3.7 The effect of PCM on NRF2 signaling pathway

**Figure 6:**
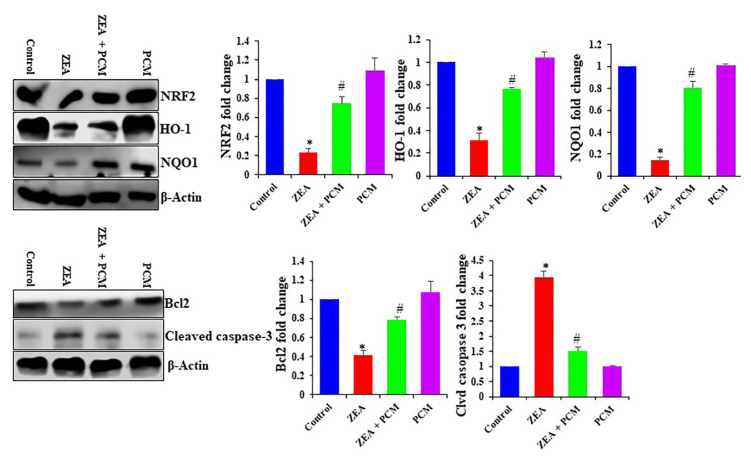
The effect of PCM on liver tissue NRF2 signaling and apoptotic proteins. (A) NRF2, HO-1, and NQO-1 expression as analyzed by Western blot, and (B) the apoptotic proteins Bcl-2 and cleaved caspase-3 as analyzed by Western blot. Significant differences compared to the control group are indicated by * (p < 0.05). Significant differences comparing the ZEA and PCM + ZEA treatment groups are indicated by # (p < 0.05).

This study aimed to clarify how PCM interacts with essential proteins in the NRF2 signaling pathway. Figure 6 shows that the levels of pNRF2, NQO-1, and HO-1 were lower in the ZEA group compared to the control group, and no substantial changes were noted in these values in the PCM-only group. However, treatment with PCM significantly elevated the expression of these proteins relative to the ZEA group.

### 3.8. The effect of PCM on apoptotic markers

ZEA exposure significantly upregulated the proapoptotic protein cleaved caspase-3 and downregulated the antiapoptotic protein Bcl-2, indicating enhanced apoptotic activity. Accordingly, ZEA treatment markedly decreased Bcl-2 expression while increasing the activation of cleaved caspase-3. Accordingly, ZEA exposure significantly reduced Bcl2 expression and activation of cleaved caspase-3. Conversely, PCM treatment increased Bcl2 expression in hepatic tissues (Figure 6). The activation of caspase-3 and the dysregulation of Bcl2 are thought to be key mechanisms by which PCMs confer protection to the liver against cell death induced by ZEA. Caspase-3, a crucial mediator of apoptosis, facilitates DNA degradation, aggregation of genetic material, and generation of apoptotic cell fragments. Thus, ZEA likely induces DNA damage and apoptosis via the activation of caspase-3 cleavage. Figure 6B shows that ZEA caused a significant increase in caspase-3 activation in hepatic tissue, while PCM inhibited it.

## 4. Discussion

ZEA is a well-documented hepatotoxic mycotoxin, and the present study provides comprehensive biochemical, histological, and molecular evidence supporting its detrimental effects on hepatic function. The experimental ZEA exposure resulted in a significant elevation of serum liver enzymes, including ALT, AST, ALP, and LDH, which are sensitive indicators of hepatocellular membrane disruption and compromised liver integrity. The observed increase in these enzymes reflects leakage into the circulatory system due to ZEA-induced hepatocyte damage, consistent with earlier reports describing ZEA-mediated hepatic dysfunction and cytotoxicity (Rajendran et al., 2020; Ahmed et al., 2022; Qi et al., 2023). Importantly, administration of PCM alone did not cause significant alterations in serum ALT, AST, ALP, or LDH levels when compared with the control group, indicating that PCM itself is not hepatotoxic. These findings confirm the hepatic safety of PCM at the administered dose and support the findings that the biochemical improvements in the ZEA + PCM group are attributable to it having protective, rather than confounding, effects. Oxidative stress plays a central role in ZEA-induced liver injury as demonstrated by the marked increase in hepatic MDA levels accompanied by a pronounced decline in endogenous antioxidant defenses, including SOD, CAT, and GPx. These biochemical alterations indicate excessive ROS generation and impaired detoxification capacity in hepatocytes. Similar reductions in antioxidant enzyme activity following ZEA exposure have been reported previously and are closely associated oxidative damage to lipids and proteins. Restoration of SOD, CAT, and GPx activities following PCM coadministration indicates the strong antioxidant capacity of PCM and its ability to reestablish redox homeostasis. The observed increase in proinflammatory cytokines, such as IL-6, IL-1β, and TNF-α, which are known to contribute to both inflammation and apoptosis in the liver, supports this finding (Meng et al., 2022). The intricate relationship between oxidative stress, inflammation, and apoptosis in ZEA-mediated hepatotoxicity is highlighted by the combined impact of these alterations. Crucially, the results of the study demonstrate how PCM protects against ZEA-induced liver damage. The ZEA-induced increases in hepatic MDA and serum liver enzymes were considerably reduced by co-administration of PCM, which also restored the activities of antioxidant enzymes. The protective effects of PCM were further validated by histopathological analysis, which showed reduced liver damage. These findings are consistent with earlier research that documented PCM’s antiinflammatory and antioxidant qualities in a range of experimental models (Shen et al., 2019). Activation of the NRF2 signaling pathway appears to be at least partially responsible for the protective effects of PCM. It has been demonstrated that exposure to ZEA causes a reduction in the expression of NRF2 and its downstream target genes, such as HO-1 and NQO1, which are essential for cellular protection against oxidative stress. This downregulation was successfully reversed by PCM coadministration, indicating that PCM can increase NRF2 expression and activity. The observed decrease in oxidative stress and the recovery of antioxidant enzyme activity are probably due to this activation of the NRF2 pathway. It has been demonstrated that activating the NRF2 pathway, a crucial regulator of cellular antioxidant defenses, provides protection against a variety of toxic insults, including mycotoxins (Obrador et al., 2023; Xiong et al., 2024). Additionally, it was revealed that ZEA exposure caused the proapoptotic protein cleaved caspase-3 to be upregulated and the antiapoptotic protein Bcl-2 to be downregulated, indicating the activation of apoptotic pathways. In addition to its effects on the NRF2 pathway, PCM altered the expression of proteins involved in the PI3K/Akt signaling pathway. According to pertinent studies, the PI3K/Akt pathway is a crucial regulator of cell survival, proliferation, and apoptosis. In the current study, PCM affected the expression of proteins in this pathway when administered concurrently with ZEA, which may have contributed to the observed antiapoptotic effects. Further research is necessary to determine the precise mechanism by which PCM modulates the PI3K/Akt pathway, but the results indicate that this pathway may also be involved in PCM’s protective effects against ZEA-induced hepatotoxicity. A notable finding is the observed decrease in proinflammatory cytokines (IL-6, IL-1β, and TNF-α)) in the presence of PCM. Downregulation of these cytokines, which is essential for the inflammatory response, indicates that PCM has strong antiinflammatory properties when addressing ZEA-induced liver damage. By reducing the inflammatory cascade that leads to tissue damage, PCM’s antiinflammatory properties may be a part of its overall hepatoprotective action. In summary, this study offers strong proof that PCM shields albino mouse livers from ZEA-induced hepatotoxicity. A number of mechanisms, such as the activation of the NRF2 signaling pathway, which lowers oxidative stress and strengthens antioxidant defenses, and possibly the modulation of the PI3K/Akt pathway, which may contribute to its antiapoptotic effects, are probably responsible for PCM’s protective effects. Based on these results, PCM may be used as a treatment to lessen the hepatotoxic effects of ZEA exposure.

PCM alleviates ZEA-induced hepatotoxicity through coordinated regulation of the PI3K/AKT/NRF2 axis and apoptosis-related proteins, particularly Bcl-2 and cleaved caspase-3. ZEA-induced liver injury is closely associated with excessive oxidative stress, which suppresses PI3K/AKT signaling and impairs NRF2 activation. Inhibition of these protective pathways results in reduced transcription of antioxidant and antiapoptotic genes, including Bcl-2.

PCM administration markedly counteracted these effects by restoring PI3K/AKT signaling, which plays a pivotal role in cell survival. Activation of AKT promotes phosphorylation-dependent stabilization of Bcl-2 while concurrently inhibiting proapoptotic signaling cascades, thereby limiting caspase-3 activation. In parallel, PCM enhances NRF2 nuclear translocation, which leads to upregulation of endogenous antioxidant defenses and attenuation of oxidative stress, a key upstream trigger of apoptosis. The combined activation of PI3K/AKT and NRF2 pathways by PCM creates a cytoprotective environment, sustains Bcl-2 expression, and suppresses apoptosis due to cleaved caspase-3.

Consistent with previous studies on natural compounds, such as curcumin and resveratrol, which have been shown to modulate Bcl-2 and caspase-3 primarily through antioxidant-dependent mechanisms (Guo et al., 2024; Noor et al., 2024), PCM exhibits a more comprehensive mode of action by concurrently targeting upstream survival signaling PI3K/AKT, NRF2, and downstream apoptotic actors. This multilevel regulation highlights PCM’s superior efficacy in restoring the balance between pro- and antiapoptotic factors.

Overall, these findings underscore PCM’s strong hepatoprotective potential against ZEA-induced toxicity by mitigating oxidative stress and inhibiting apoptotic pathways. Such findings support its promise as a complementary therapeutic strategy for toxin-induced liver injury. Future studies should investigate the protective effects of PCM in human hepatic cell lines to validate its cytoprotective mechanisms against ZEA-induced toxicity at the cellular level. To comprehensively elucidate the precise mechanisms underlying PCM’s protective effects and to validate its efficacy across human and diverse animal models, further investigation is warranted. In particular, future studies should explore the long-term impact of ZEA exposure and evaluate the potential of PCM as a preventive and therapeutic agent against chronic liver diseases associated with mycotoxin contamination.

In addition to these considerations, the following section discusses complementary mechanistic and translational aspects of PCM action that further support its therapeutic potential.

## 5. Conclusion

PCM substantially alleviates ZEA-induced hepatotoxicity in albino mice by causing a reduction in oxidative stress, inflammation, and apoptosis. Treatment with PCM reinstated antioxidant enzyme activity, corrected liver histoarchitecture, and caused a reduction in proinflammatory and apoptotic indicators. The protective effects are probably facilitated by the activation of the NRF2/HO-1/NQO1 antioxidant pathway and the modification of the PI3K/Akt signaling cascade. These data indicate that PCM possesses a therapeutic potential for mycotoxin-induced liver injury due to its antioxidant and antiinflammatory effects.

## Supplementary File

**Table S:** Antibody details of the proteins used in the immunoblot analysis.

Antibody	Company catalog	Dilution
TNF-α	(PA5-19810) Thermo Fisher Scientific, Inc. (Waltham, MA, USA)	1:2000
IL-1β	(PA5-20109) Thermo Fisher Scientific, Inc. (Waltham, MA, USA)	1:2000
IL-6	(PA5-46943) Thermo Fisher Scientific, Inc. (Waltham, MA, USA)	1:1000
pPI3K	(PA5-104853) Thermo Fisher Scientific, Inc. (Waltham, MA, USA)	1:1000
PI3K	(MA5-34900) Thermo Fisher Scientific, Inc. (Waltham, MA, USA)	1:5000
pAKT	(PAKT-140AP) Thermo Fisher Scientific, Inc. (Waltham, MA, USA)	1:1000
AKT	(44-1140G) Thermo Fisher Scientific, Inc. (Waltham, MA, USA)	1:5000
NRF2	(PA5-27882) Thermo Fisher Scientific, Inc. (Waltham, MA, USA)	1:2000
HO-1	(PA5-77833) Thermo Fisher Scientific, Inc. (Waltham, MA, USA)	1:2000
NQO1	(MA5-16098) Thermo Fisher Scientific, Inc. (Waltham, MA, USA)	1:2000
Bcl-2	(PA5-27094) Thermo Fisher Scientific, Inc. (Waltham, MA, USA)	1:2000
Cleaved caspase-3	(9661) Cell Signaling Technology, Inc. (Danvers, MA, USA)	1:3000
Secondary antibodies goat antirabbit	(AB-M-010) MOLEQULE-ON (New Lynn, Auckland, New Zealand)	1:5000
Secondary antibodies goat antimouse	(AB-M-009) MOLEQULE-ON (New Lynn, Auckland, New Zealand)	1:5000
β-actin	(MA1-91399) Thermo Fisher Scientific, Inc. (Waltham, MA, USA)	1:5000

## Data Availability

The data that support the findings of this study are available on request from the author.
